# SARS-CoV-2-associated gastrointestinal and liver diseases: what is known and what is needed to explore

**DOI:** 10.1186/s43066-021-00123-6

**Published:** 2021-07-31

**Authors:** Dina Sweed, Eman Abdelsameea, Esraa A. Khalifa, Heba Abdallah, Heba Moaz, Inas Moaz, Shimaa Abdelsattar, Nadine Abdel-Rahman, Asmaa Mosbeh, Hussein A. Elmahdy, Eman Sweed

**Affiliations:** 1 Pathology Department, National Liver Institute, Menofia University, Shibin El Kom, 32511 Egypt; 2 Hepatology and Gastroenterology Department, National Liver Institute, Menofia University, Shibin El Kom, Egypt; 3 Radiology Department, Faculty of Medicine, Menofia University, Shibin El Kom, Egypt; 4 Clinical Pathology Department, National Liver Institute, Menofia University, Shibin El Kom, Egypt; 5 Microbiology Department, Faculty of Medicine, Menofia University, Shibin El Kom, Egypt; 6 Epidemiology and Preventive Medicine Department, Menofia University, Shibin El Kom, Egypt; 7 Clinical Biochemistry, and Molecular Diagnostics Department, National Liver Institute, Menofia University, Shibin El Kom, Egypt; 8 Ohio State College of Medicine, Columbus, OH USA; 9 Biochemistry Department, Faculty of Science, Cairo University, Giza, Egypt; 10 Clinical Pharmacology Department, Faculty of Medicine, Menofia University, Shibin El Kom, Egypt

**Keywords:** COVID-19, Gastrointestinal, Liver, Pathophysiology, SARS-CoV-2

## Abstract

**Background:**

The pandemic of COVID19 which is caused by severe acute respiratory syndrome coronavirus 2 (SARS-CoV-2) was first described in China as an unexplained pneumonia transmitted by respiratory droplets. Gastrointestinal (GI) and liver injury associated with SARS-CoV-2 infection were reported as an early or sole disease manifestation, mainly outside China. The exact mechanism and incidence of GI and liver involvement are not well elucidated.

**Main body:**

We conducted a PubMed search for all articles written in the English language about SARS-CoV-2 affecting the GI and liver. Following data extraction, 590 articles were selected. In addition to respiratory droplets, SARS-CoV-2 may reach the GI system through the fecal-oral route, saliva, and swallowing of nasopharyngeal fluids, while breastmilk and blood transmission were not implicated. Moreover, GI infection may act as a septic focus for viral persistence and transmission to the liver, appendix, and brain. In addition to the direct viral cytopathic effect, the mechanism of injury is multifactorial and is related to genetic and demographic variations. The most frequently reported GI symptoms are diarrhea, nausea, vomiting, abdominal pain, and bleeding. However, liver infection is generally discovered during laboratory testing or a post-mortem. Radiological imaging is the gold standard in diagnosing COVID-19 patients and contributes to understanding the mechanism of extra-thoracic involvement. Medications should be prescribed with caution, especially in chronic GI and liver patients.

**Conclusion:**

GI manifestations are common in COVID-19 patients. Special care should be paid for high-risk patients, older males, and those with background liver disease.

## Background

The pandemic of the novel 2019 coronavirus disease (COVID-19) started in December 2019 with an outbreak of unexplained pneumonia in Wuhan, China. Severe acute respiratory syndrome coronavirus 2 (SARS-CoV-2) is primarily transmitted by droplets and aerosols, affecting the respiratory system [1]. In the USA, the first case of SARS-CoV-2-associated gastrointestinal (GI) symptoms had a 2-day history of nausea and vomiting, then progressed to diarrhea upon hospital admission [2]. Subsequently, many studies reported GI and liver infection as the first presentation of COVID-19 with later (or no) respiratory symptoms [3]. This review summarizes the demographic, clinical manifestation, radiological, and pathological findings in COVID-19 patients presenting with GI symptoms to elucidate the route of transmission and mechanism of injury and provide guidance on GI and hepatic treatment in COVID-19 patients.

## Main text

### Material and methods

A thorough PubMed search of all English-language articles published and in-press from December 2019 to December 2020 was done. The search terms were “COVID-19,” “SARS-CoV-2,” “gastrointestinal,” “liver,” “diarrhea,” “abdominal pain,” “nausea,” “vomiting,” “histopathology,” “radiology,” “pharmacology,” and “liver enzymes.” A total of 2160 studies were included in complete data extraction, and a final 590 papers were selected. The papers were reviewed, and data were collected and analyzed by gastroenterology and hepatobiliary specialists.

### The mode of transmission of SARS-CoV-2-induced GI and liver diseases

Although inhalation of respiratory droplets is the primary mechanism of SARS-CoV-2 transmission, fecal-oral transmission may be an additional source of GI infection, mainly in children. SARS-CoV-2 viral RNA was detected in the fecal matter for 11.2 to 33 days following viral clearance from the respiratory tract, indicating viral replication in the enterocytes and possible fecal-oral transmission [4]. Saliva and vomit were two additional routes of transmission. SARS-CoV-2 may infect the salivary gland by binding to and secreting from the angiotensin-converting enzyme 2 (ACE2) receptor. Therefore, saliva may play a role in the early stage of viral transmission [5]. The vomit also included viral particles either from nasopharyngeal fluids or the GI tract. The risk of viral transmission is positively correlated with vomiting severity [6]. Portincasa et al. suggest an unconfirmed hypothesis that the virus could translocate from the gut lumen into the liver via portal flow, negatively affecting hepatic cells [7]. SARS-CoV-2 could also reach the appendix via oropharyngeal contamination or appendicolith, resulting in bacterial translocation and appendicitis [8].

One study reported the possibility of SARS-CoV-2 transmission through breastmilk. However, the remaining studies did not find viral transmission in breastmilk, which is supported by the World Health Organization (WHO) that ensured breastfeeding was safe given proper precautions [9]. The low viral load in infected patients’ serum makes this transmission route limited or even non-existent [10].

### Mechanism of SARS-CoV-2-induced GI and liver diseases (Fig. 1)

#### Genetic factors

The presence of four primary intestinal transcription factors, caudal type homeobox transcription factor 2 (CDX2), hepatocyte nuclear factor 4 (HNF4), mothers against decapentaplegic homolog 4 (SMAD4), or GATA in the intestine, modulates the function and activity of ACE2 and transmembrane protease serine 2 (TMPRSS2). These results could explain the population’s variable susceptibility to GI symptoms and severity of SARS-CoV-2 infection [11].

**Fig. 1:**
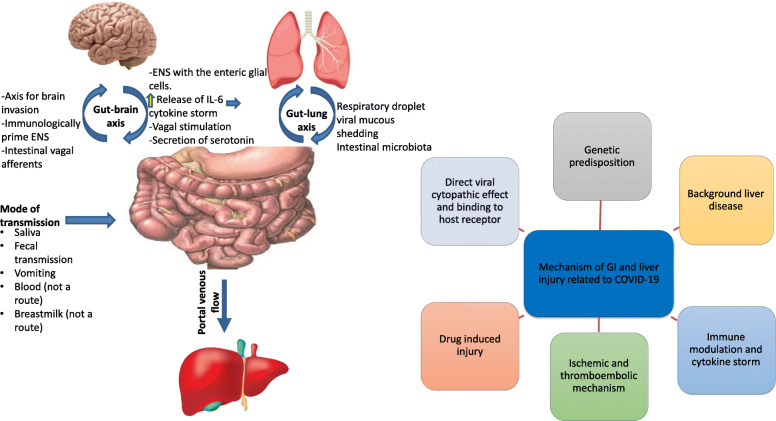
The route of spread and mechanism of SARS-CoV-2-induced gastrointestinal and liver injury. ENS, enteric nervous system; IL-6, interleukin-6

#### Central-neurological mechanism

The gut-brain axis may be a path for SARS-CoV-2 to invade the brain, ascend to the central nervous system (CNS) through intestinal vagal afferents, and immunologically prime the enteric nervous system (ENS). The ENS is strictly interconnected to the enteric glial cells (EGCs), which defend against gut pathogens by activating the Toll-like receptors (TLRs) and other inflammatory mediators. Conversely, SARS-CoV-2 neuroinvasion stimulates serotonin release and provides an alternative central mechanism for GI symptoms [12].

#### Local mechanisms

##### Viral-host receptor binding mechanism

Coronaviruses are a group of single-stranded enveloped RNA viruses that express four structural proteins: spike glycoprotein, small envelope protein, matrix protein, and nucleocapsid protein, in addition to 16 non-structural proteins [13]. SARS-CoV-2 enters host cells through the interaction between the envelope-anchored viral spike protein and the ACE2 host receptors. ACE2 receptors are widely expressed in various human cells, including the lungs, small intestine, colon, pancreatic islets, kidney, brain, vascular endothelium, and smooth and cardiac muscle [14].

**Fig. 2:**
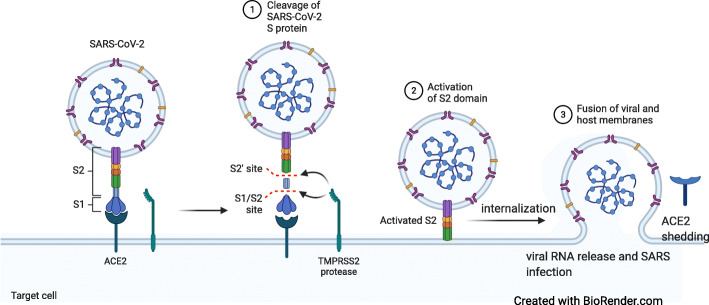
Coronavirus uses angiotensin-converting enzyme-2 (ACE2) receptor to reach the cells with the binding of the viral spike (S) glycoprotein to cellular receptors via S protein priming by host cell proteases. TMPRSS2, transmembrane protease, serine 2; SARS-CoV-2, severe acute respiratory syndrome coronavirus 2

SARS-CoV-2 has a unique structural and functional S protein that facilitates viral entry and replication. The high binding affinity of SARS-CoV-2 for human ACE2, 10–20-fold higher than SARS, increases the viral infectivity [15]. Besides, other host receptors for SARS-CoV-2, specifically CD147, TMPRSS2, endosomal cysteine proteases cathepsin B and L (CatB/L), and furin, are widely distributed in multiple organs, enhancing viral binding and entry. The mechanism of SARS-CoV-2 binding to and entering host cells is illustrated in Fig. 2.

##### Gut microbiota

The gut-lung axis is a bi-directional network in which many respiratory infections often accompany GI symptoms and vice versa. The gut microbiome plays a crucial role in modulating the immune response of SARS-CoV-2 patients to prevent vital organ damage. Alterations to the intestinal microbiota, in conjunction with an impaired immune system, contribute to COVID-19 patients’ delayed recovery and mortality [16]. Re-formulating the gut microbiota through nutritional therapy, probiotics, or fecal microbiota transplantation (using standard guidelines) may emerge as a new therapeutic target in disease management [16, 17].

##### Hypochlorhydria

Price suggests that the acidic pH of the normal gastric mucosa inactivates coronaviruses, explaining why the intestinal manifestation is more pronounced than the gastric ones [18]. However, more studies are recommended to evaluate the viral ability to survive and replicate through the extremes of GI pH.

#### Systemic mechanisms

##### Immune-related injuries

Severe SARS-CoV-2 infection results in a clinical state resembling sepsis due to the massive release of cytokines by the immune system. This cytokine storm involves innate and cellular immunity, including activation of intrahepatic CD4+ and CD8+T-cells, Kupffer cells, activation of B cells, and anti-viral antibodies. These pathways progress toward apoptosis and necrosis of infected cells, resulting in multi-organ failure late in the course of disease [19].

##### Ischemia-reperfusion injury

Severe SARS-CoV-2 cases suffer from ischemia–reperfusion injury through activation of systemic inflammatory response syndrome (SIRS), SARS, or as a complication of sepsis and hypertension [3].

##### Drug-induced GI and liver disease

Drug therapies for managing SARS-CoV-2 infection are reported to be a direct insult to the GI and liver in variable doses and conditions, discussed in a later section.

##### Symptoms and signs of GI injury in COVID-19

In a series of > 20,000 hospitalized patients in the UK, the main symptoms of COVID-19 were respiratory (71.6%), followed by enteric symptoms (nausea, abdominal pain, vomiting, and diarrhea) (29%), with 4% of patients complaining of enteric symptoms alone [20]. Patients with GI symptoms experiencing a prolonged period between the onset of symptoms and viral clearance were more likely to have a positive stool test for the virus (73.3% compared to 14.3% with no GI symptoms, p = 0.033) [21]. The prevalence of diarrhea was 5–10.3%, nausea and vomiting 5.2–11.7%, abdominal pain 2.7–8.8%, and loss of appetite 15.8%, with few reported cases of GI bleeding [22, 23].

There is no unique esophageal symptom associated with SARS-CoV-2 except for heartburn. Heartburn needs a standard treatment approach with H2 receptor antagonists or proton pump inhibitors (PPIs) [24]. Anorexia is one of the most frequent symptoms reported in COVID-19 patients, mainly associated with malaise and systemic inflammation [25].

Diarrhea may have been the only presentation in 16% of cases [26]. The diarrhea symptoms might last from 1 to 14 days with a mean of 5.4 ± 3.1 days, and the majority of patients experiencing self-limited diarrhea [21]. The possible mechanisms are direct interaction with ACE2 receptors or medications [26].

Epigastric pain, stomachache, and abdominal discomfort have been used to describe abdominal pain [27], and such pain may be a sign of gut nerve inflammation and precede the respiratory symptoms or be the sole manifestation of COVID-19. However, there was no reported data on the quality or nature of the pain characteristic of COVID-19.

Gastrointestinal bleeding in patients with COVID-19 is not as common as other GI symptoms, with a frequency ranging from 4 to 13.7%. A review of 2023 patients with COVID-19 reported only two GI bleeding cases across 15 studies [28]. The cause of bleeding is often not determined, and most patients were treated conservatively. Lower GI bleeding has also been reported in association with COVID-19, necessitating urgent consultation [29]. Ischemia may be the result of thrombotic dysfunction, hypoperfusion, and direct inflammatory effect on GI mucosa.

The prevalence of acute pancreatitis was 0.27% among hospitalized COVID-19 patients with no other identifiable etiology [30].

### Laboratory findings on COVID-19-associated GI and liver injury

**Fig. 3:**
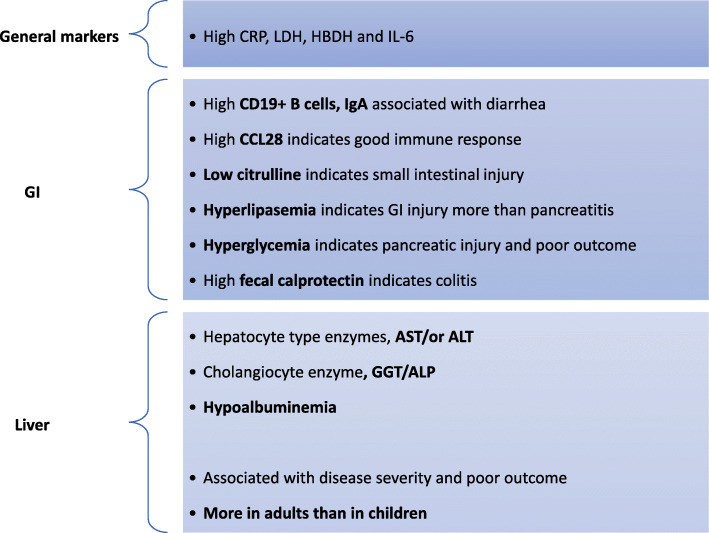
Laboratory findings in COVID-19 patients presented with GI or liver affection. CRP, C-reactive protein; LDH, lactate dehydrogenase; HBDH, α-hydroxybutyrate dehydrogenase; CCL28, chemokine (C-C motif) ligand 28; GGT, gamma-glutamyl transferase; AST, aspartate aminotransferase; ALT, alanine aminotransferase; ALP, alkaline phosphates; IL-6, interleukin-6

Figure 3 illustrates the most common laboratory tests specific for GI and liver infection with their possible clinical implications.

#### General systemic and inflammatory markers (not organ-specific)

COVID-19 patients showed high levels of C-reactive protein (CRP), lactate dehydrogenase (LDH), and α-hydroxybutyrate dehydrogenase (HBDH) as a result of inflammation-inducing GI or liver injury [31]. Interleukin-6 (IL-6) is a crucial cytokine contributing to the host defense by producing acute-phase proteins and proliferation of B-lymphocytes and neutrophils. In the liver, SARS-CoV-2 activates hepatic stellate cells and Kupffer cells to produce many inflammatory factors such as tumor necrosis factor-α (TNF-α), interleukin (IL-6), and chemokines [19]. Moreover, COVID-19 patients with chronic liver disease (CLD) had a higher serum IL-6 level than those without CLD [32].

#### Laboratory findings specific for GI infection

A high proportion of serum CD19+ B cells, IgA, and low citrulline have been reported in COVID-19 patients presenting with diarrhea, indicating a direct viral cytopathic effect or intestinal ischemia inducing mucosal injury [33]. In contrast, increased serum CCL28 expression was associated with a good mucosal immune response [34]. Fecal calprotectin (FC) has evolved into a reliable biomarker allowing detection of intestinal inflammation in inflammatory bowel disease (IBD) and infectious colitis. High FC in COVID-19 patients indicates an acute GI inflammatory response and/or serves as a potential indicator of the progressive course in IBD patients [35].

Although SARS-CoV-2 induced pancreatic injury, elevated lipase levels exceeding three times the upper limit could be an alarm for GI injury rather than pancreatic injury. However, hyperlipidemia is not considered a marker of severe COVID-19 infection or a poor clinical outcome [36]. Acute hyperglycemia and transient type-2-diabetes also indicate a pancreatic injury and are associated with poor prognosis.

#### Laboratory findings specific for liver infection

The available liver enzyme data varied widely due to a lack of a standard cut-off point and a consensus definition of severe cases. The prevalence of liver abnormalities has been classified into hepatocyte-type (aspartate aminotransferase [AST]/alanine aminotransferase [ALT]) (20.75%), cholangiocyte-type (gamma-glutamyl transferase [GGT]/alkaline phosphatase [ALP]) (29.25%), and mixed types [37]. According to the frequency of elevated hepatocyte-type markers, ALT and AST were found to be elevated in 21.2–25% and 15.2–25% of patients, respectively. AST is typically located in the cytosol and mitochondria of hepatocytes, mainly in zone 3. Therefore, elevated serum AST could reflect direct cytopathic damage of hepatocytes or hypoxic changes. However, AST is not a specific marker for liver injury and has a broader organ distribution, indicating its involvement in multi-organ damage [38].

The prevalence of cholangiocyte-related enzymes showed increased levels of GGT (22.7%) and total bilirubin (9%), though serum ALP is controversial. Since GGT and ALP are expressed in sites other than the bile duct, their level could indicate multi-organ damage and cannot be classified solely as a specific bile duct marker. Furthermore, GGT has been identified as a surrogate marker for increased oxidative stress and chronic inflammation [39]. Therefore, elevated cholangiocyte markers are not specific for bile damage, as shown by rare pathological features of bile damage or cholestasis.

The synthetic liver function is altered by SARS-CoV-2 infection, resulting in hypoalbuminemia, an independent predictor of patient mortality. Albumin deficiency may be related to insufficient protein intake, serum protein exudation due to inflammation, diarrhea, or direct liver injury [40]. Furthermore, hepatic dysfunction badly impacts the coagulative and fibrinolytic pathways, platelet count, neutrophil counts with high neutrophil-to-lymphocyte ratios, and serum ferritin levels [33].

### Radiological findings during the SARS-CoV-2 pandemic

Radiology plays a fundamental role in diagnosing COVID-19 patients based on chest findings. rRT-PCR may give initial false-negative results with a sensitivity of 83.3% for early COVID-19. In contrast, typical CT radiological findings demonstrated a sensitivity of nearly 97.2% for diagnosing early COVID-19 and a low rate of missed COVID-19 diagnoses [41, 42]. However, chest CT should not be used exclusively for diagnosing COVID-19 infection, especially in asymptomatic patients [43]. Moreover, abdominal imaging becomes critical in COVID-19 patients who initially or exclusively present with GI symptoms. Abdominal imaging plays a role in determining the mechanism of SARS-CoV-2-induced injury, which may be thromboembolic or non-thromboembolic.

### Abdominal imaging findings in patients with SARS-CoV-2 infection

**Fig. 4:**
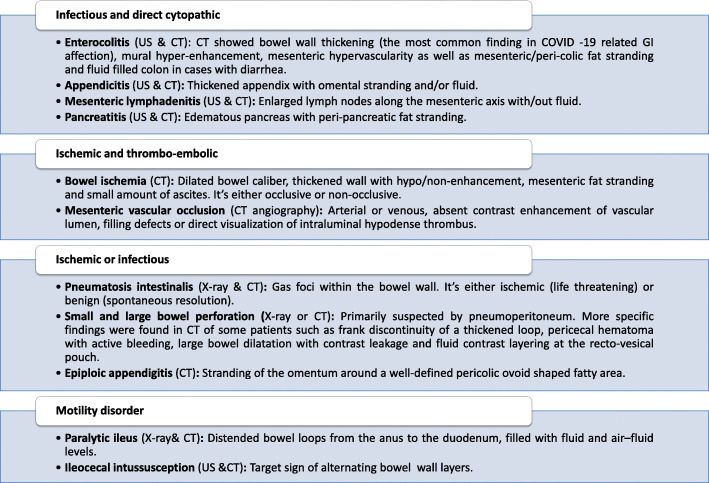
Abdominal radiological findings of COVID-19-associated GI symptoms. CT, computed tomography; US, ultrasound; GI, gastrointestinal

Numerous radiological findings related to COVID-19 have been reported. The findings were separated into four groups to clarify the potential cause of injury (see Fig. 4).

Using contrast-enhanced computed tomography (CECT), enterocolitis of the abdomen was mainly observed in the right-sided colon (ascending colon and transverse colon) but was also involved in any bowel loop [44]. Many bowel ischemia cases in either the small or large bowel were detected by CECT. Occlusive ischemia resulted from thrombosis of the large or small mesenteric arteries, as determined by CT angiography or pathology, respectively. However, non-occlusive bowel ischemia with patent mesenteric arteries was attributed to hypoxia or low cardiac output. In CT angiography, the presence of isolated venous thrombosis in the mesenteric, portal vein, or inferior vena cava was associated with bowel wall edema without evidence of ischemic changes [45]. Pneumatosis intestinalis, a radiological sign suggestive of necrotizing enterocolitis, is another characteristic associated with bowel ischemia in COVID-19 patients. However, pneumatosis intestinalis might be discovered accidentally in the absence of clinical or radiological evidence of bowel ischemia. Epiploic appendagitis and perforation of the small and large bowel have been reported in some cases as direct sequelae of infection or secondary to ischemia [44].

Motility disorders have been identified in few COVID-19 patients, with some exceptions; one case with paralytic ileus resolved after conservative treatment [46]. Another case involved a pediatric patient presenting with abdominal pain and ileocecal intussusception [47]. Motility disorder could be induced by an imbalance of the colon’s autonomic innervation, a common feature of coronaviruses. However, further research is recommended to determine if these findings are linked to COVID-19 or concomitant.

Two studies reported the association of appendicitis and mesenteric lymphadenitis with SARS-CoV-2 infections. However, further ancillary studies on the resected specimen were recommended to confirm this hypothesis [8]. Similarly, a COVID-19 case has been identified in which a patient presented with a mild form of pancreatitis, absent of other causes [44].

### Hepatic imaging findings in patients with SARS-CoV-2 infection

Hepatic injury was reported frequently in abdominal ultrasound (US) and CT in the form of hepatomegaly, periportal edema, pericholecystic fat stranding, and portal lymphadenopathy. One study showed a significantly lower liver to splenic CT attenuation ratio in COVID-19 patients than in the control group. Assessment of liver stiffness (LS) using combined US and elastography revealed a linear correlation between LS and biochemical markers for acute liver damage, indicating that elastography can be used as a reliable non-invasive indicator of hepatic injury in COVID-19 patients [48].

Hepatic steatosis appearing as a hepatic hypodensity in CT was higher in COVID-19 patients than in the control group. Thus, hepatic steatosis was suggested as a risk factor for SARS-CoV-2 infection [44]. However, it is unknown whether steatosis is a risk factor or a result of COVID-19.

Biliary system manifestations were reported in COVID-19 patients in the form of bile stasis with subsequent gall bladder distention and sludge formation [44]. Furthermore, edema of the gall bladder wall, hepatic bed, and pericholecystic fat stranding were observed independently or as part of hepatic injury.

Finally, solid organ acute infarction was reported in two COVID-19 patients, involving the kidney, spleen, or liver [44].

### Histopathological findings of COVID-19-associated GI and liver injury

The role of histopathology in the diagnosis of COVID-19 aims to detect viral host receptors, assess pathological features, visualize viral particles, and understand disease mechanisms. Moreover, virus detection by immunohistochemistry (IHC) can be recognized directly by the pathologist, saving time and money to confirm a potential suspected case of COVID-19.

### Pathological findings of GI-related COVID-19

The small intestine, primarily absorptive and crypt enterocytes but not goblet, Paneth, or enteroendocrine cells, expressed more ACE2 receptors than other GI sites [49]. Furthermore, histopathological analysis of 14 GI specimens obtained either post-mortem or by resection revealed ischemic changes in the form of mucosal necrosis or transmural hemorrhagic infarction [50]. The possible etiology of ischemia was thrombi in mucosal or mesenteric blood vessels in six cases, vasculitis and endothelial inflammation in four cases, and mixed thrombi and endotheliitis in two cases. Hobnail modifications of the endothelial cells with bizarre nuclear shapes have been also reported [51]. Inflammation was evident in seven cases: two of lymphoplasmacytic nature, two of acute nature, and three of undefined nature. Three cases of microscopic colitis were reported; however, there is insufficient evidence to determine whether these cases are SARS-CoV-2 related or incidental findings. There were seven attempts to detect SARS-CoV-2 viral particles in GI specimens. Positive viral particles were observed in the mucosa and the endothelial cells in five and two cases, respectively [51]. Moreover, Stah et al. detected intact viral particles in the bowel endothelium 8 weeks after initial infection and viral clearing in respiratory and blood specimens [52]. However, negative results were reported in six cases: two for the esophagus, two for the colon, one for the stomach, and one for the duodenum. The two reported appendicitis cases showed mucosal necrosis, non-caseating granulomas, and a foreign body reaction associated with severe mesenteric necrotizing lymphadenitis or ulcerophelgmonous [8].

### Pathological findings of liver-related COVID-19

There has been a limited pathology role in diagnosing liver-associated COVID-19 abnormalities, with the majority of specimens obtained post-mortem [32, 53–55]. Histopathological characteristics varied, with most cases exhibiting a mixed pattern of injury. Histopathological changes included steatosis, either microvesicular and/or macrovesicular (61/95), inflammation with variable distribution including portal, sinusoidal, lobular, or panacinar (64/101), vascular abnormalities (54 cases), endotheliitis (3 cases), and lobular necrosis/apoptosis (15 cases). Other pathological findings reported in low frequency included Kupffer cell hyperplasia, giant cells transformation, lobular cholestasis, granuloma, and type II fibrinogen deposition. Moreover, bile duct damage was reported in only two cases. Numerous experiments used various techniques to visualize the viral particles, including electron microscopy (EM), polymerase chain reaction (PCR), or even IHC, with positive findings encountered in 15/22 cases [54, 55]. Rather than viral particles, some authors interpreted the EM findings as clathrin-coated vesicles, which are involved in synaptic vesicle reconstitution, or multi-vesicular bodies, which are routinely discovered post-mortem. Several studies have conducted a C4d IHC test to assess immunological background, and only one case was positive (1/50) [54–56].

**Fig. 5:**
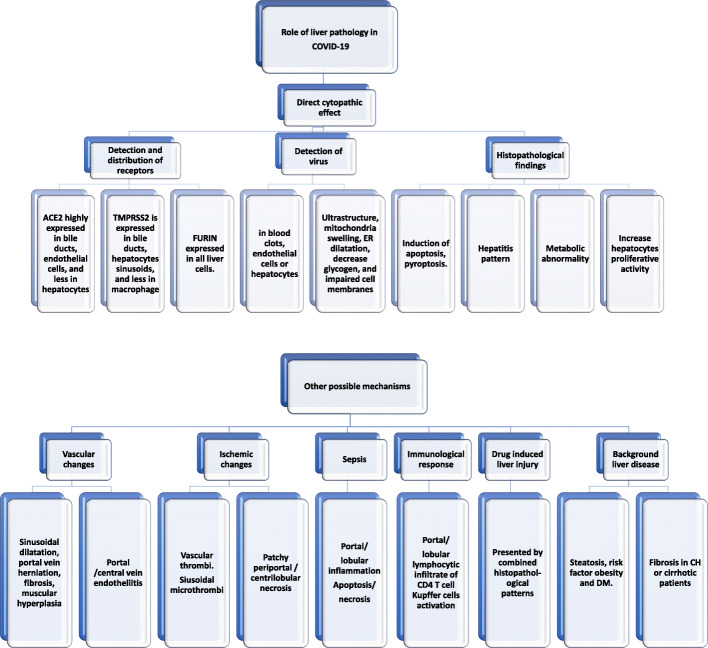
The role of pathology in understanding SARS-CoV-2-induced liver injury mechanism. ACE2, angiotensin-converting enzyme-2; TMPRSS2, transmembrane protease, serine 2

The histopathological findings of steatosis, acute hepatitis, and positive viral tissue detection indicate a poor patient outcome. Histopathological observations were interpreted to gain a better understanding of the potential mechanism of injury (see Fig. 5).

### Management therapies in SARS-CoV-2-associated GI and liver diseases

Treatment of COVID-19 associated GI or liver infection aims to clear the viral infection, relieve symptoms, and stabilize patients with previous GI or liver diseases. All therapies are critical for their anti-inflammatory properties and reduce viral entry, host receptor binding, and replication.

In addition to viral target therapies, symptomatic treatment is discussed, including oral or intravenous hydration and antiemetic medications. The anti-diarrheal agent loperamide can be used in patients without fever or bloody stools and after ruling out other infectious causes. Patients with non-variceal upper GI bleeding can be conservatively treated with PPIs and coagulation optimization without endoscopic intervention. However, PPIs are associated with hypochlorhydria, which increases the risk of SARS-CoV-2 entering the gut from the stomach, causing viral infection. Therefore, PPIs should be used at the lowest effective dose (once daily) [57]. Similarly, endoscopic evaluation for lower GI bleeding might be initially postponed until acute disease resolution. For patients with severe COVID-19-associated liver damage, hepatoprotective, anti-inflammatory, and jaundice-reducing agents, as well as vitamin E, are recommended [58].

### Management of background GI and liver diseases during the SARS-CoV-2 pandemic

Many studies discussed the strategies for managing IBD patients during the SARS-CoV-2 pandemic in connection with several factors, mainly proper interpretation of the complaint, whether related to COVID-19 or acute flaring of the primary disease. For active IBD patients without SARS-CoV-2 infection, adding or escalating anti-inflammatory or biologic therapy for symptomatic improvement and remission induction may be involved. However, systemic glucocorticoids should be used at the lowest effective therapeutic dose [59]. On the other hand, the aim of therapy in patients with inflammatory bowel disease (IBD) infected with SARS-CoV-2 is to minimize immunosuppression during active viral infection to avoid viral complications (e.g., pneumonia). Two strategies are proposed for COVID-19 patients with IBD in remission: continue therapy indefinitely with budesonide, aminosalicylates, sulfasalazine, topical glucocorticoids, and antibiotics [59], or temporarily adjust medication until symptoms resolve, including systemic glucocorticoids and immunomodulators [60].

Immunosuppressive therapy reduction or discontinuation is not recommended for asymptomatic patients who have undergone liver transplantation and post-transplant treatment unless they are SARS-CoV-2 positive. Similarly, HCC-related treatments should be administered without any delay.

### Drug-induced GI and liver injury (DILI)

Certain drugs are alleged to play a role in GI and liver injury. The possible mechanisms include reactive metabolites and oxidative stress, idiosyncratic through drug-cytochrome P-450 interaction, or synergistic inflammatory response [61–63]. Table 1 summarizes the potential therapeutic agents, their mechanism of action, common side effects relating to the GI and liver, and possible drug-drug interactions (DDIs).

**Table 1:** Potential GI and liver adverse effects and drug interaction profile of COVID-19 investigational drugs

	Mechanism of action	GI affection	Liver affection	Major drug-drug interactions
Chloroquine/hydroxychloroquine [80]	Interferences with terminal glycosylation of ACE2 receptor Blocks viral entry by increasing endosomal pH and inhibiting viral fusion to the cell membrane	Nausea, vomiting, weight loss, abdominal pain	Rare elevations in aminotransferases. Most reactions are Idiosyncrasy or oxidative stress.	A moderate inhibitor of CYP2D6 and P-gp Significant particularly with anti-rejection immunosuppressants. Weak interaction with tenofovir/entecavir Hydroxychloroquine given to a patient taking hepatitis c treatment should monitor for cardiac arrhythmia
Ivermectin [81]	Inhibition of viral IMPα/β1-mediated nuclear import, which reduces the replication of the virus and so the viral load	Nausea, vomiting, diarrhea	Very few reports on elevated liver enzymes or Jaundice	Avoid concomitant use of ivermectin with other drugs that enhance GABA activity
Nitazoxanide [82]	Antiparasitic drug has broad-spectrum anti-viral activity	Abdominal pain (8%), diarrhea (2%), nausea (3%), vomiting (1%)	Increased ALT: <1%	Rapidly hydrolyzed to tizoxanide. which is highly protein-bound (>99%), so caution when giving with other highly protein-bound drugs with narrow therapeutic indices.
Atazanavir [83]	Protease inhibitors	Diarrhea, nausea, vomiting, abdominal pain	Indirect hyperbilirubinemia with overt jaundice Elevation of hepatic enzymes especially in patients with underlying HBV or HCV co-infection	Inhibitor of CYP3A4 and CYP2C9 PPI decreases its concentrations. Tenofovir and efavirenz should not be co-administered with atazanavir
Favipiravir [84]	RNA-dependent RNA polymerase inhibitor	Nausea/vomiting (5–15%), diarrhea (5%)	Liver enzyme abnormalities	Inhibitor for: CYP2C8 and aldehyde oxidase
Interferon beta [85]	Cytokines with anti-viral and immunomodulatory effects.	Nausea, vomiting	Elevated liver enzymes	DDI potential not fully evaluated. Possible inhibitor of CYP enzymes
Lopinavir/ritonavir [86]	HIV protease inhibitor/CYP450inhibitor	Nausea/vomiting (5–10%), abdominal pain (1–10%), diarrhea (10–30%), dysgeusia (< 2%), increased serum amylase/lipase.	Hepatotoxicity ranges from mild elevations in aminotransferases to acute liver failure. Recovery takes 1–2 mo. Might include drug-cytochrome P-450 interaction	Substrate for: CYP3A4, CYP2D6, P-gp Inducer for: CYP1A2, CYP2B6, CYP2C8, CYP2C9, CYP2C19, UGT1A1 Inhibitor for: CYP3A4 Increased levels of Immunosuppressive drugs (calcineurin and mTOR inhibitors). Moderate interaction risk with tenofovir with renal functions monitoring. Lopinavir/ ritonavir increase concentrations of hepatitis C treatment.
Remdesivir [87]	RNA-dependent RNA polymerase inhibitor	Nausea, vomiting	Deranged liver enzymes Hepatotoxicity reported; frequency is not yet known.	NA
Ribavirin [88]	Inhibit capping of viral messenger RNA, and the viral RNA-dependent polymerase	Nausea	Hepatotoxicity	NA
Anakinra [89]	IL-1R inhibitor	Rare abdominal pain, nausea, diarrhea	Hepatobiliary disorders: Elevated transaminases, noninfectious hepatitis	No effect on CYP450.
Baricitinib [90]	JAK1 and JAK2 inhibitor Inhibit viral endocytosis	Bowel perforation, nausea, vomiting	Hepatitis B reactivation	Partially metabolized by CYP3A4 and a substrate for OAT3 and P-gp OAT3 inhibitors cause a significant effect on baricitinib exposure
Dexamethasone/ Hydrocortisone [59]	Reduction of IL-8, monocyte chemo-attractant protein-1, and Th1 chemokine IFN-γ-inducible protein-10	Nausea, peptic ulcers	NA	Aspirin can increase the risk of bleeding when used with it
Ruxolitinib [91]	Selective JAK inhibitors	NA	Increased ALT Increased AST	Metabolized by CYP3A4 and CYP2C9. So, it is liable to DDIs with inhibitors or inducers of these enzymes. Ruxolitinib may inhibit BCRP and P-gp, and caution is indicated with co-administering with substrates of these transporters with narrow therapeutic indices.
Sarilumab [92]	IL-6R inhibitor	Few cases of gastrointestinal perforation	Increased ALT	No effect on CYP450
Tocilizumab [93]	IL-6R inhibitor (Curbs cytokine release syndrome)	Bowel perforation, pancreatitis, abdominal pain	Elevated liver enzymes, Reactivation of chronic hepatitis B	No effect on CYP450

*ACE* angiotensin-converting enzyme, *ALT* alanine aminotransferase, *AST* aspartate aminotransferase, *BCRP* breast cancer resistance protein, *COVID-19* coronavirus disease-19, *CYP* cytochrome P450, *DDI* drug-drug interaction, *GABA* γ-aminobutyric acid, *GI* gastrointestinal, *HIV* human immunodeficiency virus, *IFN* interferon, *IL* interleukin, *IMP* α/β-mediated nuclear import, *JAK* Janus kinase, *OAT* organic anion transporter, *P-gp* P-glycoprotein, *PPI* proton pump inhibitor, *Th* t-helper, *TOR* target of rapamycin

Hepatic patients with non-alcoholic fatty liver disease (NAFLD) infected with SARS-CoV-2 might be more susceptible to DILI [64]. Dexamethasone was found to decrease mortality rates among COVID-19 patients; however, it may lead to chronic hepatitis B virus (HBV) reactivation. Similarly, tocilizumab, an IL-6 blocker, increases HBV reactivation risk. Therefore, hepatitis B surface antigen (HBsAg)-positive patients should also be treated with anti-viral medication for the duration of steroid therapy.

For patients with severe alcoholic or autoimmune hepatitis, caution must be taken when suggesting the initiation of steroids or other immunosuppressive therapy [65]. Regimens containing chloroquine or remdesivir were generally considered safe. Hydroxychloroquine should be treated for cardiac arrhythmias in patients receiving hepatitis C treatment [66].

### Demographic data of SARS-CoV-2-associated GI and liver infection

#### Geographical distribution of GI symptoms

The SARS-CoV-2 associated with GI manifestations was reported later in the COVID-19 pandemic. A potential reason is that the prevalence of GI symptoms is 2–3 times lower in China, the epicenter of the outbreak, than in western countries, primarily Europe and the USA; however, there was no statistically significant difference between the country-based studies [23]. Furthermore, an analysis of Chinese studies showed a constant low prevalence of diarrhea and vomiting before, during, and after April [67]. These observed differences could result from variability in SARS-CoV-2 host receptor gene expression, coagulation activity, and health care access amongst different socio-economic groups and ethnicities, all of which affect COVID-19 pathogenesis. Chinese populations have a lower risk of thrombo-embolic complications than other ethnic groups, which reduces the severity of COVID-19 [68]. However, geographic differences between countries remain unexplored.

#### Age-related GI and liver symptoms

COVID-19 patients with GI symptoms ranged in age from 1 day to 92 years, with a pooled mean age of 48.7 ± 16.5 years [39]. The frequency of patients presenting with COVID-19-related GI symptoms did not show much variance, remaining at nearly 10% for all age groups [69]. Age was positively correlated with the severity of GI symptoms and mortality. Possible factors include low expression of ACE receptors, lower intensity of viral exposure, the protective effects of live vaccines, increased susceptibility to recurrent infections, and the difference in the adaptive, cellular immunity, and microbiota in children. In contrast to the age-related vascular and endothelial damage, prior coronavirus exposure and associated comorbidities negatively impact the disease course in the elderly [70].

#### Gender differences of SARS-CoV-2-associated GI and liver symptoms

According to a recent meta-analysis by Kaur et al., which included 6635 COVID-19 patients, COVID-19-infected individuals were predominantly male. However, the manifestation of GI symptoms was significantly different between males and females. Self-reported GI symptom frequency during the COVID-19 course was significantly higher among women than men (P < 0.001). Zouh et al. found a significantly higher proportion of female COVID-19 patients with GI symptoms associated with COVID-19 [71]. The exact mechanism is not elucidated; however, it could be hormonal modulation of the gustatory system. Notably, global data suggested male gender is a negative indicator of disease severity and mortality. Factors responsible for higher male mortality could include higher rates of smoking, respiratory tract infection, proinflammatory cytokines, and the immunosuppressive effect of testosterone. However, Agrawal et al. suggested that the estrogen-enhancing effect and the localization of immune response genes on X-chromosome may protect females [72].

#### The prognosis of SARS-CoV-2-induced GI and liver infection

There was no consensus regarding the impact of GI symptoms on the COVID-19 course. Studies reported no significant difference in the prevalence of diarrhea, nausea, or vomiting between severe and non-severe patients [22]. Another study reported patients presenting with COVID-19-related GI symptoms to have a low mortality rate compared to those without GI symptoms [73]. In contrast, other studies reported a poor outcome for COVID-19 patients who presented with GI symptoms, especially abdominal pain [69, 74]. The low prognostic impact of GI symptoms could be related to the marked electrolyte imbalance, gut dysbiosis, ischemic-reperfusion injury, and associated neurological manifestations.

Notably, there was a consensus of the poor prognostic impact of elevated liver enzymes on COVID-19 patients [69]. In extreme COVID-19 patients, hypoalbuminemia, high GGT, aminotransferase (AST > ALT), and bilirubin rather than serum ALP levels were observed [39].

#### The impact of background GI and liver diseases on the outcome of SARS-CoV-2 infection

There was no correlation between autoimmune GI diseases and the increased risk of SARS-CoV-2 infection. Patients with IBD are not at greater risk and can maintain remission with maintenance therapy [59]. Similarly, celiac disease patients showed no increased risk for SARS-CoV-2 infection or primary disease complications [75].

In CLD patients, SARS-CoV-2 infection stimulates inflammatory mediators and decreases ACE2 expression, aggravating liver cirrhosis [76]. Furthermore, NAFLD patients infected with SARS-CoV-2 might be more vulnerable to DILI, a cytokine storm, and ischemic damage to the liver [77]. Similarly, HBV patients coinfected with SARS-CoV-2 could experience HBV reactivation following therapy [78]. Therefore, the liver function in patients with CLD should be monitored regularly throughout the SARS-CoV-2 infection.

## Discussion

The digestive tract may serve as a possible route of SARS-CoV-2 transmission to the liver, appendix, and brain. The virus reaches the gut through fecal transmission, saliva, or vomiting. The persistence of viral indicators in the stool may be used as a surrogate monitor for recurrent infection [27]. A GI and liver injury mechanism is a multi-hit hypothesis requiring interaction between genetics, multiple organ cross-talk, and vascular and inflammatory response [4]. Demographic studies have proposed a low prevalence of COVID-19-associated GI symptoms in China compared to other countries; however, there is no consensus on gender or age as predicting factors [27]. The high-risk factors include the male gender, old age, anorexia, abdominal pain, liver enzyme abnormalities, and the histopathological findings of steatosis, acute hepatitis, and positive viral tissue detection. Surprisingly, SARS-CoV-2 infection may worsen the background liver disease, with low or no impact on pre-existing GI diseases [76].

Typical GI symptoms experienced by some COVID-19 patients included diarrhea, nausea, vomiting, and abdominal pain, which may even necessitate surgical interventions [20, 29]. Therefore, all patients with GI symptoms should be eligible for SARS-CoV-2 testing as these symptoms may precede the respiratory symptoms or be the only symptoms [23]. In contrast, liver injury is observed during laboratory investigation or post-mortem pathological studies. Liver function tests should be monitored even in the absence of hepatic symptoms. Abnormalities in liver enzymes are reported in similar frequencies despite the presence of pre-existing liver disease [79], and unexplained elevation of ALT/AST, an increase of bilirubin, and reduced albumin levels in a clinically suspect patient may indicate COVID-19 infection. The mechanism of GI injury is multifactorial, with infection and ischemic-thromboembolic alteration playing a significant role. A subsequent intestinal malabsorption, imbalance in intestinal secretions, intestinal dysbiosis, and activation of the enteric nervous system exacerbate the GI infection. Even more, the mucosal injury could mediate viral spread throughout the bowel wall [26].

Similarly, the mechanism of liver injury is multifactorial, confirmed by mixed histopathological findings, laboratory results, and radiological investigations. The direct viral cytopathic effect has been explained by broad hepatic SARS-CoV-2 host receptor distributions [15]. Vascular alterations result from increased blood flow, endothelial injury, endotheliitis, thrombosis, and concomitant thrombotic changes in the pulmonary vessels [54]. However, Lagana et al. reported that liver vascular abnormalities do not correlate with pulmonary dysfunction [55]. The lack of pathological features of ischemia did not explain the elevated serum aminotransferase levels in COVID-19 patients but rather raises the possibility of another mechanism other than hypoxic-ischemic injury. Similarly, the pathological findings of sepsis are reported only in four cases [55]. The immunological and cytokine storm mechanism contributes significantly to liver damage by releasing inflammatory mediators and activating different immune cells [19]. The expression of C4d was focally positive in only one case [54, 55]. Therefore, the immunological response is more cellular than humoral. DILI is a possible mechanism, though it is still a diagnosis of exclusion. The presence of steatosis and mixed histopathological findings supports drug-induced damage [55]. However, Wang et al. found no significant differences in drug adherence between patients with normal and abnormal liver enzymes [22]. Moreover, hepatotoxicity usually occurs after long-term antiviral therapy. Finally, most histopathological changes in the liver were limited or related to underlying liver diseases [53].

### Suggestions for future research

COVID-19 associated with GI and liver injury is an emerging research era with many questions with no definite answers. The mechanism by which some individuals experience GI symptoms rather than respiratory symptoms is not well elucidated. The route of GI transmission and the virus’s ability to survive the extremes of GI pH require further studies. The reliable role of elevated liver enzymes during SARS-CoV-2 infection must also be investigated because liver damage is usually an incidental finding in the routine laboratory or pathological investigation. Improving the histopathological tools in detecting viral particles could aid in elucidating disease mechanisms. More studies are recommended to identify the essential structural proteins of SARS-CoV-2 that promote tissue invasion and replication. Accordingly, improved selective and targeted therapeutic agents could be developed.

## Conclusions

SARS-CoV-2 infection resulted in GI and liver disease through multi-hit complex mechanisms. GI manifestations are normal in COVID-19 patients, and particular attention should be given to high-risk patients, including those who are older, male, and have abdominal pain, elevated liver enzymes, or background liver disease.

## Data Availability

Available from the corresponding author on reasonable request.

## Abbreviations

SARS-CoV-2: Severe acute respiratory syndrome coronavirus 2
GI: Gastrointestinal
COVID-19: Novel 2019 coronavirus disease
ACE2: Angiotensin-converting enzyme 2
TMPRSS2: Transmembrane protease serine 2
ENS: Enteric nervous system
EGCs: Enteric glial cells
TLRs: Toll-like receptors
CatB/L: Cathepsin B and L
SIRS: Systemic inflammatory response syndrome
PPIs: Proton pump inhibitors
CRP: C-reactive protein
LDH: Lactate dehydrogenase
HBDH: α-hydroxybutyrate dehydrogenase
IL-6: Interleukin-6
TNF-α: Tumor necrosis factor-α
CLD: Chronic liver disease
FC: Fecal calprotectin
IBD: Inflammatory bowel disease
AST: Aspartate aminotransferase
ALT: Alanine aminotransferase
GGT: Gamma-glutamyl transferase
ALP: Alkaline phosphatase
CECT: Contrast-enhanced computed tomography
US: Ultrasound
LS: Liver stiffness
EM: Electron microscopy
PCR: Polymerase chain reaction
IHC: Immunohistochemistry
DDI: Drug-drug interactions
DILI: Drug induced liver injury
HBV: Hepatitis B virus
HBsAg: Hepatitis B surface antigen
NAFLD: Non-alcoholic fatty liver disease
